# Evaluación del análisis de la relación albúmina-creatinina mediante tira reactiva como método de cribado para la determinación de albúmina en orina en atención primaria

**DOI:** 10.1515/almed-2025-0102

**Published:** 2025-08-15

**Authors:** Paula Liébanas García, Silvia Montolio Breva, Teresa Sans Mateu

**Affiliations:** Laboratori Clínic ICS Camp de Tarragona – Terres de l’Ebre – Hospital Tortosa Verge de la Cinta, Tarragona, España

**Keywords:** albuminuria, prueba de detección, tiras reactivas, enfermedad renal crónica

## Abstract

**Objetivos:**

Las técnicas cuantitativas son el método de referencia más habitual en la determinación de la albúmina en orina, una pieza fundamental en el diagnóstico y seguimiento de la enfermedad renal crónica (ERC). Por otro lado, se han desarrollado métodos semicuantitativos, como las tiras reactivas de orina, con resultados alentadores como herramientas de cribado rápidas y eficaces y con una buena relación coste-beneficio. El objetivo de este estudio era evaluar la viabilidad de la tira reactiva de Meditape UC-11A (Sysmex, Kobe, Japón) como herramienta para detectar la albuminuria, frente a los métodos cuantitativos que determinan la relación albúmina-creatinina (ACR).

**Métodos:**

Se analizaron sistemáticamente 1.627 muestras empleando las tiras Meditape UC-11A cuando se solicitaba la cuantificación de albúmina en orina. Comparamos la ACR obtenida con la tira reactiva con la obtenida con el método convencional (método cuantitativo de Atellica, Siemens, Marburg, Alemania) y calculamos los indicadores diagnósticos. Así mismo, evaluamos las implicaciones económicas de emplear las tiras, teniendo en cuenta los precios de las pruebas (2,88€ en el caso del método cuantitativo para la albúmina y la creatinina, y 0,93€ en el caso de la tira Meditape UC-11A).

**Resultados:**

Se realizó un análisis de la curva ROC para determinar el valor óptimo de corte, con el que se obtuvo una tasa de concordancia del 90,78 %. Utilizando la prueba cuantitativa como método de referencia, la tira Meditape UC-11A mostró una sensibilidad y especificidad, valor predictivo positivo (VPP), y valor predictivo negativo (VPN) para ACR de 84,2 %, 91,9 %, 62,7 %, y 97,3 %, respectivamente. Estos resultados indican un ahorro potencial del 81,2 % con respecto a las pruebas cuantitativas durante el periodo de estudio, lo que hubiera supuesto un ahorro de 2291,65€.

**Conclusiones:**

Los resultados obtenidos respaldan la recomendación de emplear las tiras Meditape UC-11A para la detección de niveles alterados de albuminuria, lo que supone una alternativa viable a los métodos cuantitativos, permitiendo un ahorro de costes significativo.

## Introducción

La enfermedad renal crónica (ERC), caracterizada por la pérdida gradual de la función renal, se ha convertido en un problema de salud pública de primer orden, habiéndose publicado guías de práctica clínica para mejorar los resultados clínicos de los pacientes con esta patología [1], [2].

La ERC viene definida por la presencia de alteraciones en la estructura o función renal que persisten durante un periodo superior a los tres meses [3]. Dichas alteraciones son: 1) tasa de filtración glomerular (TFG) inferior a 60 mL/min/1,73 m^2^; 2) albuminuria; 3) anomalías en el sedimento urinario, histología o imágenes de daño renal; 4) trastornos tubulares renales; o 5) historia de trasplante renal.

Aunque la ERC se clasifica en cinco estadios en función de la TFG, no existe un consenso sobre los valores de corte de la TFG, lo que se ha intentado solventar realizado diversos meta-análisis [4]. A la luz de los resultados de dichos meta-análisis, a la TFG se le añadió el nivel de albuminuria, y el estadio 3 de TFG se subdividió en 3a y 3b para reflejar con mayor precisión el pronóstico. Por otro lado, se ha observado una relación entre el estadio de ERC y el riesgo cardiovascular, estando los estadios más avanzados asociados a un mayor riesgo de desarrollar enfermedad cardiovascular [5].

La albúmina, la proteína más abundante en la orina en los pacientes sanos, es el marcador de daño renal y ERC de uso más extendido [6]. Así, la presencia de albuminuria predice el desarrollo de daño renal en los primeros estadios de la enfermedad, pudiendo por sí sola causar daño tubulointersticial, al activar los mediadores proinflamatorios [7].

La determinación de la albúmina presenta algunas ventajas con respecto a la determinación de proteínas totales en orina: no solo presenta una mayor exactitud y precisión, sino que también es un marcador más eficaz de la pérdida de proteínas secundaria a alteraciones en la filtración glomerular [8].

El método de referencia para detectar la liberación de proteínas a través del riñón es un método cuantitativo en una muestra de orina de 24 horas. Esta medida muestra una excelente correlación con la relación albúmina-creatinina (ACR) medida en la primera orina de la mañana, resolviendo algunas de las limitaciones de larecogida de orina de 24 horas. De este modo, la ACR facilita la obtención de la muestra de orina y reduce la variabilidad resultante tanto del estado de hidratación del paciente como del ritmo circadiano.

Un valor de ACR inferior a 30 mg/g se considera normal; mientras que un valor de entre 30 y 300 mg/g indica albuminuria moderada, y los valores superiores a 300 mg/g son indicativos de albuminuria grave [1].

Aunque los métodos cuantitativos son considerados el método de referencia, la albuminuria, la creatinuria y, por tanto, la ACR también se pueden detectar fácilmente aplicando técnicas semicuantitativas como las tiras reactivas.

Los últimos avances tecnológicos han supuesto un importante paso adelante en la automatización del urianálisis. La adopción de la tecnología de semiconductores complementarios de óxido metálico (CMOS) ha permitido mejorar la sensibilidad analítica, habiendo mostrado potencial para la detección de la microalbuminuria [9], [10]. Además, tanto el análisis de partículas en la orina por microscopía como su alternativa, la citometría de flujo, representan un gran avance en este campo.

Diversos estudios han demostrado la eficacia de estas técnicas semicuantitativas [11], [12], [13], [14] como posibles herramientas de detección, siendo estas más rápidas y económicas que los métodos cuantitativos, a expensas de una menor sensibilidad.

Los pacientes con diabetes e hipertensión presentan un mayor riesgo de desarrollar ERC. En 2014, el 8,5 % de la población con una edad superior a los 18 años padecía diabetes, una cifra que se espera vaya en aumento [15], [16]. Por otro lado, se estima que, en el año 2000, más de una cuarta parte de la población adulta presentaba hipertensión, una cifra que se habrá incrementado en un 60 % para el año 2025 [17], [18].

Según las guías de práctica clínica [19], a la población con mayor riesgo de desarrollar ERC se les debería realizar al menos un cribado al año, con el fin de identificar a los pacientes con un estadio inicial de ERC y prevenir su progresión, así como el desarrollo de daño cardiovascular [20], [21].

Dado que en los próximos años se prevé un aumento tanto de la enfermedad renal crónica como de las enfermedades que predisponen a desarrollarla, y teniendo en cuenta la elevada demanda de pruebas de albúmina para poblaciones muy diferentes de pacientes, resulta imperativo contar con un método de detección económico, rápido y con suficiente sensibilidad para hacer frente al incremento de la carga de trabajo y reducir costes y tiempos de respuesta.

Nuestra hipótesis de trabajo es que el análisis de ACR mediante el uso de tiras reactivas se podría emplear como prueba de detección semicuantitativa en muestras de orina de pacientes de atención primaria, lo que reduciría la realización de pruebas cuantitativas, derivando en una reducción en el consumo de recursos, y acortaría los tiempos de respuesta cuando el resultado es negativo.

El objetivo principal de este estudio es evaluar la viabilidad de emplear tiras reactivas para la semicuantificación de la ACR como prueba de cribado en las muestras procedentes de atención primaria. Así mismo, se analiza la relación coste-beneficio de esta nueva estrategia, comparando el uso de las tiras reactivas con la cuantificación de la albúmina y la creatinina en orina.

## Materiales y métodos

### Contexto y pacientes

Las muestras de orina se procesaron en el laboratorio clínico ubicado en el Hospital de Tortosa Verge de la Cinta (HTVC), un hospital de agudos perteneciente al Servicio Catalán de Salud, que asiste a una población de 179.891 pacientes.

Aunque el laboratorio clínico recibe muestras de pacientes ambulatorios y hospitalizados, en el estudio únicamente se incluyeron las muestras de pacientes adultos atendidos en atención primaria.

### Diseño del estudio

Se realizó un estudio retrospectivo unicéntrico y transversal en el HTVC, entre los meses de octubre y noviembre de 2023. El estudio fue aprobado por el Comité Ético del Institut d’Investigació Sanitària Pere Virgili (IISPV).

### Obtención de datos

Se recogieron muestras de orina espontánea en tubos sin aditivos siguiendo el procedimiento habitual del hospital para su posterior procesamiento la misma mañana en el laboratorio del HTVC.

### Métodos analíticos

Se cuantificó la albúmina mediante inmunoturbodimetría con PEG (Atellica, Siemens, Marburg, Alemania). La muestra que contiene albúmina se diluye y posteriormente se hace reaccionar con un antisuero específico para formar un precipitado que puede medirse por turbidimetría a 340/596 nm.

La cuantificación de la creatinina se realiza siguiendo el método multienzimático basado en la técnica desarrollada por Suzuki y Yoshida (Atellica, Siemens, Marburg, Alemania). Una vez realizadas las reacciones, se genera un compuesto colorimétrico cuya absorbancia se puede medir a 545/694 nm.

El análisis con las tiras reactivas Meditape UC-11A se llevó a cabo utilizando el equipo UC-3500 (Sysmex, Kobe, Japón). Las tiras presentan tres almohadillas: una almohadilla de detección de creatinina basada en el método Benedict-Behre (con un intervalo de medición de entre 10 y 300 mg/dL) y dos almohadillas de medición de albúmina basada en el principio de error proteico del azul de tetrabromofenol, un indicador de pH. La diferencia entre las dos almohadillas es la cantidad de tetrabromofenol azul: la primera contiene 10 μg de tetrabromofenol azul (con un intervalo máximo de albúmina de 0,150 g/L) y la segunda 5 μg de tetrabromofenol azul para concentraciones de albúmina superiores a 10 g/L. La concentración de albúmina se mide en 10, 30, 80, 150 o>150 mg/dL, mientras que la de creatinina se mide en 10, 50, 100, 200 o 300 mg/dL. Para realizar el control de calidad interno, previamente al inicio del estudio se evaluó la precisión intraensayo e interensayo utilizando material de control UC-Control High Level y UC-Control Low Level (Sysmex, Kobe, Japón).

Los valores de ACR se calcularon dividiendo la concentración de albúmina entre la concentración de creatinina obtenida tanto con la prueba de Atellica como con la tira reactiva. Estos resultados se encuentran disponibles, previa petición al autor para correspondencia.

### Estudio transversal

En este estudio retrospectivo, comparamos la ACR cuantitativa obtenida mediante una plataforma de análisis químico (Siemens, Marburg, Alemania) y la obtenida con una prueba semicuantitativa en la que se leyeron las tiras reactivas en un sistema automatizado (Sysmex, Kobe, Japón) utilizando muestras de primera hora de la mañana de 1.627 pacientes.

Para realizar este estudio, se realizó un análisis semicuantitativo de la ACR en todos los pacientes de atención primaria para los que se solicitó la cuantificación de la albúmina en orina. Primero, se realizó un análisis semicuantitativo de cada muestra utilizando el equipo UC-3500  y, a continuación, se realizó la cuantificación con el autoanalizador de Atellica (Siemens, Marburg, Alemania).

La recogida de datos se realizó entre octubre y noviembre de 2023. A continuación, exportamos a una hoja Excel de Microsoft la determinación cuantitativa de la albúmina y la creatinina, así como la relación albúmina-creatinina, paralelamente a los tres resultados por cada tira reactiva (ACR, albúmina y creatinina).

### Análisis estadístico

Calculamos la sensibilidad, especificidad, valor predictivo positivo (VPP) y valor predictivo negativo (VPN) de la tira reactiva. Se generaron curvas ROC empleando los paquetes pROC de R [22] para determinar el valor óptimo de corte [23], siguiendo el procedimiento descrito en estudios anteriores [24], [25].

Para identificar el valor de corte más eficaz, calculamos el índice de Youden (J). Este índice se suele emplear en la evaluación de pruebas diagnósticas, dado que optimiza el equilibrio entre la sensibilidad y la especificidad, facilitando así la identificación del valor de corte con el mejor rendimiento diagnóstico global.

La normalidad de la distribución de los datos se comprobó mediante el test de Kolmogorov-Smirnov. A partir de estos resultados, calculamos el coeficiente de correlación de Spearman para evaluar la concordancia entre los valores ACR obtenidos con la tira reactiva y con el método cuantitativo [26]. Así mismo, aplicamos la prueba de Chi cuadrado para comparar la frecuencia de los resultados positivos y negativos de ACR obtenidos con los dos métodos de medición, empleando la ACR obtenida con el método cuantitativo como la técnica de referencia, donde un valor <30 mg/g indicaba un resultado negativo.

La significación estadística se estableció en p<0,05 y los análisis se llevaron a cabo con R Commander.

En el análisis de coste-beneficio, calculamos el ahorro potencial comparando el coste de determinar cuantitativamente la albúmina y la creatinina (2,88€) con el coste de hacerlo utilizando la tira Meditape UC-11A (0,93€).

## Resultados

Se incluyó en el estudio a 1.627 participantes, de los que el 49 % eran mujeres y el 51 % eran hombres, con una edad media de 66 años (con un intervalo de entre 15 y 99 años). En todas las muestras se determinó la ACR tanto con la tira reactiva como con el método cuantitativo, que se estableció como el estándar de referencia. La tira reactiva Meditape UC-11A, analizada con el sistema UC-3500, mostró una precisión satisfactoria en cuanto a variabilidad intra e interensayo, al probarla con el material de control.

En la Tabla 1 se presenta la distribución de los niveles de albúmina y creatinina en las 1.627 muestras analizadas con la tira reactiva Meditape UC-11A. Como se puede observar, la tira Meditape UC-11A suele subestimar los niveles de albúmina y sobreestimar los niveles de creatinina, especialmente a concentraciones elevadas.

**Tabla 1:** Distribución de albúmina y creatinina determinadas con la tira Meditape UC-11A.

	Meditape UC-11A	Análisis cuantitativopi
	n	Mediana (intervalo)
Albúmina, mg/L		29,3 (3–2160,3)
10	1,312	5 (3–45,2)
30	168	28,1 (3,1–75,7)
80	66	67,7 (14,2–134)
105	2	215 (203,9–226,8)
150	79	398 (8,4–2160,3)
Creatinina, mg/dL		97,3 (7,9–452,6)
10	83	24,4 (7,9–78,2)
50	543	52,1 (27,5–125,4)
100	556	94,9 (60,4–226)
200	374	156,5 (97,8–304,2)
300	71	235,4 (155,7–452,6)

**Figura 1:**
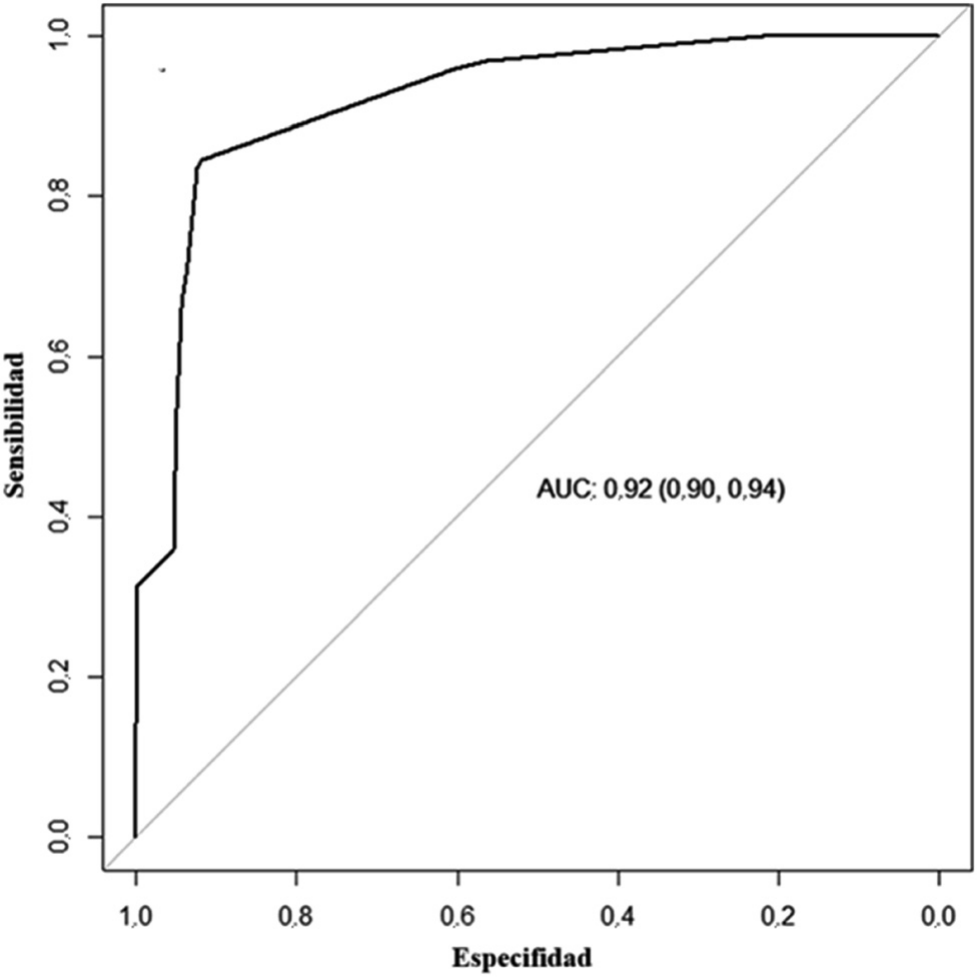
Curva ROC para la determinación de la ACR con la tira reactiva Meditape UC-11A.

Para evaluar la exactitud diagnóstica de los valores de ACR obtenidos con la tira reactiva, generamos una curva ROC, con la que se obtuvo un área bajo la curva (AUC) de 0,92 (IC 95 %: 0,90–0,94), lo que indica una capacidad de discriminación excelente a la hora de detectar alteraciones en la ACR (Figura 1).

El valor de corte óptimo para la determinación de la ACR con las tiras reactivas se estableció en 26,67 mg/g, mostrando una sensibilidad del 84,2 % y una especificidad del 91,9 %. Este fue el valor de corte con el que se obtuvo el índice de Youden más alto (J=0,76), lo que confirma su solidez a la hora de optimizar el rendimiento de la prueba (Tabla Suplementaria S1).

Tras analizar la curva ROC, se comparó la frecuencia de los resultados de ACR positivos y negativos obtenidos en las 1.627 muestras analizadas con la tira reactiva con los obtenidos con método cuantitativo de referencia (Tabla 2). De todas las muestras analizadas con la tira Meditape UC-11A, la ACR fue ≥26,67 mg/g en 306 muestras (18,81 %) y <26,67 mg/g en 1.321 muestras (81,19 %). Por el contrario, en la prueba cuantitativa, la ACR fue ≥30 mg/g en 228 muestras (14.01 %) y <30 mg/g en 1.399 (85,99 %). La prueba de Chi cuadrado reveló una asociación significativa (X^2^=742,76, valor p<2,2 × 10^−16^), lo que demuestra la coherencia entre los resultados de los dos métodos. Los parámetros de exactitud diagnóstica de la tira Meditape UC-11A, esto es, sensibilidad, especificidad, valor predictivo positivo (VPP) y valor predictivo negativo (VPN) se detallan en la Tabla 3.

**Tabla 2:** Comparación de la ACR determinada con la tira reactiva Meditape UC-11A con los valores obtenidos con la prueba cuantitativa.

	Atellica, Siemens	
Meditape UC-11A (Sysmex)	≥30 mg/g	<30 mg/g
≥26,67 mg/g	192	114
<26,67 mg/g	36	1,285

**Tabla 3:** Sensibilidad, especifidad, VPP y VPPN de la ACR analizada con UC-3500, aplicando el valor de corte de 26,67 mg/g.

	ACR Meditape UC-11A (IC 95 %)
Sensibilidad, %	84,2 (78,9–88,4)
Especifidad, %	91,9 (90,3–93,2)
VPP, %	62,7 (57,2–68)
VPN, %	97,3 (96,3–98)

Así mismo, analizamos la relación entre los valores de ACR obtenidos con la tira reactiva y los obtenidos con el método cuantitativo. Dado que en la prueba de Kolmogorov-Smirnov los datos no mostraron una distribución normal, aplicamos el coeficiente de correlación de Spearman, que indicó una correlación significativa y consistente entre los dos métodos (rho de Spearman=0,82, valor p<2,2 × 10^−16^). El elevado nivel de concordancia obtenido confirma la fiabilidad de la tira reactiva como herramienta de cribado, ya que los resultados de esta eran coincidentes con los de la prueba cuantitativa, por lo que puede emplearse para la determinación de los niveles de albúmina en orina en pacientes de atención primaria.

Con la tira reactiva, se obtuvieron 36 falsos negativos en los resultados de ACR (2,21 %), que oscilaron entre los 30,16 y los 116,18 mg/g (mediana: 46,47) en las 1.627 muestras. Por otro lado, se obtuvieron 114 falsos positivos (7,01 %) en los resultados de ACR de la tira reactiva, cuyos niveles oscilaron entre los 2,4 y los 29,10 mg/g (mediana: 8,16).

En total, se podrían haber evitado 1.321 (81,2 %) cuantificaciones de albúmina en orina, lo que hubiera derivado en un ahorro hipotético de 2.291,65 euros durante el periodo de estudio. Si se hubiera empleado la tira reactiva durante un año, esto habría supuesto un ahorro de 20.601,72 euros.

## Discusión

Realizamos un estudio para evaluar la eficacia de las tiras reactivas Meditape UC-11A a la hora de detectar albuminuria en pacientes de atención primaria. La curva ROC mostró un AUC excepcional (superior a 0,9), lo que indica la notable capacidad de la prueba para identificar alteraciones en la ACR.

El índice de Youden nos permitió establecer el valor de corte óptimo con el fin de garantizar un correcto equilibrio minimizando los falsos positivos y los falsos negativos. Este valor de corte no solo mejora la exactitud diagnóstica de la prueba, sino que evidencia su fiabilidad y posible utilidad como herramienta de cribado en la práctica rutinaria.

Los resultados de este estudio demuestran la existencia de una asociación significativa, así como una correlación fiable entre los valores de ACR obtenidos con la tira Meditape UC-11A y los obtenidos con el método cuantitativo. Estos hallazgos subrayan la utilidad de la tira reactiva como herramienta de cribado fiable y accesible para las poblaciones sin un diagnóstico previo de enfermedad renal. La capacidad de esta prueba para detectar los primeros síntomas de daño renal la convierten en un valioso recurso en atención primaria, permitiendo una intervención precoz y, por ende, mejorando los resultados clínicos.

Dado que el objeto del presente estudio era evaluar la capacidad de detección de Meditape UC-11A, resulta fundamental analizar el número de falsos negativos. Así, la tasa de falsos negativos fue del 2,21 %. En todos estos casos, los valores fueron inferiores a 116 mg/g, siendo inferiores a 50 mg/g en el 70 % de las muestras. Estos resultados se podrían deber a la tendencia de la tira Meditape UC-11A a subestimar los niveles de albúmina y a sobreestimar los valores de creatinina, especialmente a concentraciones altas. Sin embargo, un seguimiento estrecho en este tipo de casos no debería representar un problema para el control posterior de la enfermedad.

Al determinar el valor de corte aplicando la curva ROC, pudimos reducir el número de falsos positivos. Sin embargo, aun aplicando este valor de corte optimizado, seguíamos obteniendo una tasa de falsos positivos del 7,01 %. Al analizar con detenimiento estos falsos positivos, observamos una tendencia significativa: existía una proporción considerable de ellos en los que la ACR se había calculado aplicando el método cuantitativo y se acercaba mucho al valor de corte establecido. Así mismo, en los casos en los que la tira reactiva de orina indicó un valor de creatinina de 10 mg/dL, el resultado de la ACR era consistentemente positivo, mientras que el método cuantitativo únicamente confirmó el 21,68 % de los resultados positivos. Esta discrepancia indica una posible limitación de las tiras reactivas a la hora de medir con exactitud los niveles de creatinina, especialmente a concentraciones bajas.

A pesar de las limitaciones inherentes a las pruebas de cribado, el bajo porcentaje alcanzado de falsos positivos y falsos negativos demuestra un rendimiento aceptable. Estos porcentajes se ven influidos principalmente por la sensibilidad de la técnica, propia de su naturaleza como prueba de cribado. De este modo, el bajo porcentaje observado de falsos positivos y falsos negativos es indicativo del éxito del estudio, ya que se cumple el objetivo principal de reducir el número de cuantificaciones.

Los resultados obtenidos indican que el método de cribado consistente en la utilización de tiras reactivas posee una sensibilidad del 84,2 % y una especificidad del 91,9 %, con un VPP y un VPN del 62,7 % y el 97,3 %, respectivamente. Sin embargo, en estudios previos en los que se evaluó el mismo tipo de tiras reactivas, la sensibilidad, especificidad, VPP y VPN para la detección de albuminuria (UACR>30 mg/g) fueron del 97-97,5 %, 44–67 %, 22–70,3 % y del 97,1–99 % respectivamente [11], [12]. Esta variabilidad podría atribuirse a la diversidad de las poblaciones de estudio, los criterios de inclusión y exclusión aplicados, las metodologías empleadas para la cuantificación de ACR y a diferencias en los valores de corte.

Con respecto a la implementación de una nueva estrategia de trabajo en el laboratorio, nuestro estudio sugiere que la utilización de tiras reactivas como método de cribado, seguida de la cuantificación únicamente en las muestras donde se obtuvo un resultado positivo sería una alternativa con una buena relación coste-beneficio. Según muestra nuestro análisis, la aplicación de esta estrategia evitaría la cuantificación de aproximadamente el 80 % de los análisis de albúmina en orina solicitados, lo que redundaría en un ahorro significativo de costes.

Además, la tira semicuantitativa de ACR ofrece diversas ventajas con respecto a la tecnología cuantificativa al tratarse de un proceso de determinación rápido que aprovecha los protocolos de laboratorio existentes para el urianálisis. En general, no solo resulta de utilidad para ahorrar costes, sino que también libera personal y tiempo, para que puedan dedicarse a tareas de mayor relevancia.

No obstante, aunque los resultados obtenidos son prometedores, este estudio no está exento de algunas limitaciones. Se debería realizar estudios para validar los hallazgos realizados y determinar el impacto clínico y económico a largo plazo de esta estrategia de detección. Así mismo, el posible ahorro identificado podría no traducirse de forma generalizada en otros países o contextos, dado que nuestro laboratorio pertenece al Sistema Público de Salud.

En conclusión, los resultados obtenidos subrayan el potencial de la tira Meditape UC-11A para la determinación de la ACR como una valiosa herramienta de cribado para la detección de alteraciones en la ACR en pacientes de atención primaria. El robusto AUC, sumado al valor de corte óptimo identificado demuestran la utilidad clínica de esta estrategia para la detección y el manejo precoz de los desórdenes relacionados con la función renal. La implementación de esta estrategia no solo evitaría la realización de cuantificaciones innecesarias en el futuro, sino que también supondría un ahorro considerable de costes, si se aplicara en la práctica habitual.

## Supplementary Material

Supplementary Material

## Material Suplementario

Este artículo incluye material suplementario (https://doi.org/10.1515/almed-2025-0102).
